# The Advantage of Supine and Standing Heart Rate Variability Analysis to Assess Training Status and Performance in a Walking Ultramarathon

**DOI:** 10.3389/fphys.2020.00731

**Published:** 2020-07-24

**Authors:** Martina Anna Maggioni, Lea Christiane Rundfeldt, Hanns-Christian Gunga, Marc Joerres, Giampiero Merati, Mathias Steinach

**Affiliations:** ^1^Charité – Universitätsmedizin Berlin, Corporate Member of Freie Universität Berlin, Humboldt-Universität zu Berlin, and Berlin Institute of Health, Institute of Physiology, Center for Space Medicine and Extreme Environments, Berlin, Germany; ^2^Department of Biomedical Sciences for Health, Università degli Studi di Milano, Milan, Italy; ^3^IRCCS Fondazione Don Carlo Gnocchi, Milan, Italy

**Keywords:** heart rate, heart rate variability, autonomic modulation, ultramarathon, exercise, low intensity

## Abstract

Cardiac autonomic modulation of heart rate, assessed by heart rate variability (HRV), is commonly used to monitor training status. HRV is usually measured in athletes after awakening in the morning in the supine position. Whether recording during standing reveals additional information compared to supine remains unclear. We aimed to evaluate the association between short-duration HRV, assessed both in the supine and standing position, and a low-intensity long-duration performance (walking ultramarathon), as well as training experience. Twenty-five competitors in a 100 km walking ultramarathon underwent pre-race supine (12 min) and standing (6 min) HR recordings, whereas performance and subjective training experience were assessed post-race. There were no significant differences in both supine and standing HRV between finishers (*n* = 14) and non-finishers (*n* = 11, mean distance 67 km). In finishers, a slower race velocity was significantly correlated with a higher decrease in parasympathetic drive during position change [larger decrease in High Frequency power normalized units (HF_*nu*_: *r* = −0.7, *p* = 0.01) and higher increase in the detrended fluctuation analysis alpha 1 index (DFA1: *r* = 0.6, *p* = 0.04)]. Highly trained athletes accounted for higher HF_*nu*_ during standing compared to poorly trained competitors (+11.5, *p* = 0.01). Similarly, greater training volume (total km/week) would predict higher HF_*nu*_ during standing (*r* = 0.5, *p* = 0.01). HRV assessment in both supine and standing position may provide additional information on the dynamic adaptability of cardiac autonomic modulation to physiologic challenges and therefore be more valuable for performance prediction than a simple assessment of supine HRV. Self-reported training experience may reliably associate with parasympathetic drive, therefore indirectly predicting long-term aerobic performance in ultramarathon walking races.

## Introduction

Ultramarathon, defined by track lengths exceeding marathon distance (i.e., 42.195 km), has gained considerable interest over the past decades (Hoffman and Krishnan, 2013). Ultra-endurance races may account for distances up to 1,000 km and duration up to 25 days, as well as extreme environmental conditions (e.g., subarctic or desert climates, high mountains, etc.) (Rundfeldt et al., 2018; Tiller et al., 2019). Among these competitions, 24-h-long races present a particular physiologic strain, by combining prolonged exercise and sleep deprivation with possible unexpected course conditions and weather changes (Cottin et al., 2007).

Human physiologic responses to ultra-endurance exercise include neuromuscular fatigue (Millet, 2011), increased cardiac strain (Cote et al., 2015) and persistent shifts in cardiac autonomic modulation of heart rate (Gratze et al., 2005; Rundfeldt et al., 2018), the last being assessed by means of Heart Rate Variability (HRV) (Task Force of the European Society of Cardiology, and the North American Society of Pacing, and Electrophysiology, 1996; Taralov et al., 2015). Prolonged low aerobic workloads are generally correlated with an increase in resting total HRV and parasympathetic drive indices, whereas high and anaerobic workloads reduce these parameters (Aubert et al., 2003; Iellamo et al., 2004).

To date, the use of HRV for assessing the state of aerobic training and performance prediction has generated conflicting results (Buchheit, 2014). Indeed, HRV may change depending on training periodization. A “spot” evaluation of the HRV therefore has poor significance, if the current training status of the athlete and the time of the training period during which he is performing are unknown (Buchheit and Gindre, 2006; Buchheit, 2014). Furthermore, HRV indices may vary with the measurement conditions, like daytime (as HRV exhibits a typical circadian profile) (Shaffer et al., 2014), the subject recumbency during recording (supine, sitting, standing) (Fürholz et al., 2013), the RR series length (from minutes to 24 h) (Task Force of the European Society of Cardiology, and the North American Society of Pacing, and Electrophysiology, 1996), and the temporal proximity with training or competition, including possible influences like neuro-hormonal adaptation to stress (Hynynen et al., 2011) and pre-competitive anxiety (Morales et al., 2013), as well as the effect of tapering periods (Hug et al., 2014).

Currently, HRV assessment in athletes has been mostly performed in the morning upon awakening (Aubert et al., 2003). As 5-min recordings are considered the acceptable minimum, especially for spectral measurements (Task Force of the European Society of Cardiology, and the North American Society of Pacing, and Electrophysiology, 1996), 5- to 10-min long beat-to-beat HR recordings to assess HRV are usually employed. Among athletes, the supine position appears to be the most sensitive tool to monitor training status and performance (Aubert et al., 2003). However, the additional informative value of HRV recording during standing, compared to measurements exclusively in supine position, remains to be elucidated.

Indeed, the standing posture may add some valuable information (Schneider et al., 2019), as it provokes a light and persistent (order of minutes) shift in sympathovagal balance (Gonçalves et al., 2015). This autonomic shift may be influenced by training-related phenomena such as an overall vagal enhancement due to aerobic training, pre-competitive anxiety, or mere stress (Hynynen et al., 2011) and fatigue carry-over from preceding exercise sessions (Schäfer et al., 2015), as well as overtraining (Hynynen et al., 2008). Thus, autonomic adaptation to orthostatic position may be associated with both aerobic training status and performance prediction, and provide additional information beyond an exclusively supine recording, also regarding external circumstances and influences (Gratze et al., 2005; Hynynen et al., 2008, 2011; Grant et al., 2009).

Therefore, this study aimed to evaluate the relationships between short-duration HRV both assessed during supine rest (12 min) and standing (6 min), with performance and training status, in a group of amateur athletes participating in a 100 km walking ultramarathon, the “*Mammutmarsch.*” We hypothesized that the better trained and then more successful competitors would account for higher resting parasympathetic predominance pre-race. Furthermore, we assumed that the cardiac autonomic changes during standing may provide additional information on the association between HRV and performance, as well as training status, compared to recording exclusively during supine rest.

## Materials and Methods

### The *Mammutmarsch*

The *Mammutmarsch* ultramarathon annually takes place near Berlin, Germany, at the end of May. With a total distance of 100 km, the course leads through the surroundings of Berlin mostly over paved streets, as well as forest trails or dirt tracks. The racetrack is mainly flat (maximal elevation gains ≤100 m), due to the specific geographical location of the city of Berlin. The time to complete the 100 km race is limited to 24 h, and it is mandatory for participants to perform the race by walking only, which is a feature that makes the *Mammutmarsch* considerably unique. Therefore, competitors undergo a very prolonged but low-intensity exercise. Regarding the starting time, all competitors are assigned to starting groups and thus enter the race between 3 and 5 p.m. The *Mammutmarsch* is an amateur ultramarathon, with a wide range of experience and training level among the competitors.

### Study Design and Participants

This study was conducted as part of a larger investigation called *“Baseline characteristics, performance predictors and physiological changes in a 100 km walking ultramarathon.”* A call among athletes competing in the *Mammutmarsch* in the years 2015, 2016, and 2018 was distributed through information material sent out by the race organizers as well as social networks. All competitors were eligible for study inclusion. Interested participants contacted the study team and, upon acceptance to partake in the study, were personally invited to the laboratory. All volunteers provided their written and informed consent to participate, and the study was conducted in accordance with the Declaration of Helsinki and approved by the Ethics Committee of the Charité University of Medicine, Berlin, Germany (document number EA1/163/14).

**FIGURE 1:**
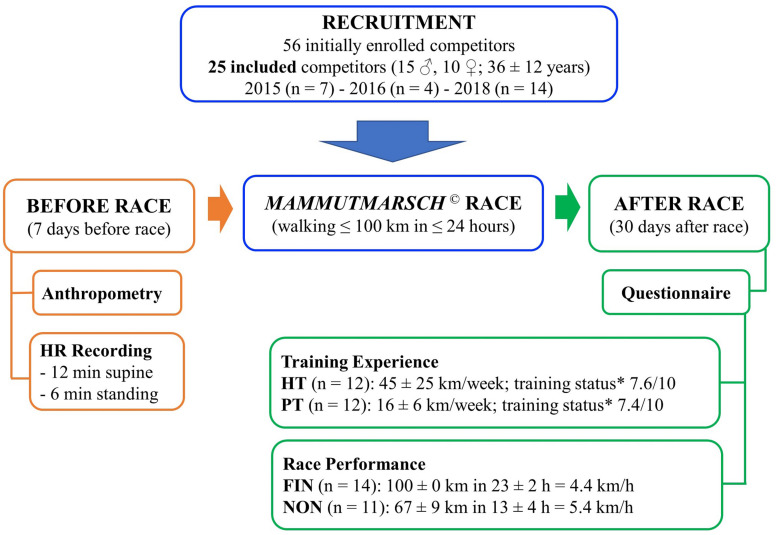
Study design. Experimental procedures overview employed in all three *Mammutmarsch* competitions of 2015, 2016, and 2018 (m ± SD). *Self-rated training status on a 10-point Likert scale.

A total of 56 competitors were enrolled in the larger investigation (2015: *n* = 20; 2016: *n* = 20; 2018: *n* = 16), and 25 of them were included also in our study (section “Statistical Analysis” and Figure 1).

#### Experimental Protocol

Study design is reported in Figure 1. Pre-race data collection was performed during the 7 days prior to the race, between 8 and 12 a.m., in a laboratory at the Charité Berlin. All participants had been advised to refrain from strenuous exercise, as well as consumption of coffee, alcohol, or other stimulants, during the 12 h before the measurement. All recordings took place in a quiet and comfortable room at constant ambient temperature (between 18 and 21°C).

After informed consent explanation and acceptance, anthropometric data were measured, then beat-to-beat HR was recorded for 12 min in supine (SUP), and, after verbal advice, for further 6 min in standing (STD) position (total duration of HR monitoring: at least 18 min). HR recordings were performed with a validated heart rate monitor (Polar Mod. RS800CX, Polar Electro Oy, Kempele, Finland) (Hernando et al., 2018). During HR recording, participants were requested to breathe normally and to avoid speaking and moving.

Moreover, on the day after the race, a questionnaire was sent to all competitors (to be returned within 30 days), to assess their individual performance in the *Mammutmarsch*, as well as training experience and subjectively rated training status (Supplementary Figure S1 and section “Performance and Training Experience”).

### Outcome Measures

#### Heart Rate Variability

All RR interval series were first visually inspected by an experienced operator. Artifacts were removed and only recordings of at least 12 min during SUP and 6 min length during STD (eligibility cut-off ≤3 % artifacts), as well as recordings exhibiting sinus rhythm (verified by visual inspection of a preliminary 12-lead standard ECG), were deemed eligible for analysis. Using a commercial software (Kubios HRV vs. 2.1, Kuopio, Finland), validated indices of HRV were assessed, as markers of cardiac autonomic modulation of heart rate, in the time-, frequency-, and non-linear domain (Task Force of the European Society of Cardiology, and the North American Society of Pacing, and Electrophysiology, 1996). According to Tarvainen et al. (2014), the filter threshold was set at the “low” level. The correction was performed by replacing the identified artifacts with interpolated values using a cubic spline interpolation (Tarvainen et al., 2014).

To standardize the analysis, the last 10- and 5-min segments were selected from each eligible recording. In the time-domain, the root mean square of successive NN (normal-to-normal) differences (RMSSD) was computed, which represents vagal drive. As for frequency-domain, the spectral power of NN oscillation in the high frequency (HF: 0.15–0.40 Hz) band was assessed and reported also as normalized units (HF_*nu*_) (Task Force of the European Society of Cardiology, and the North American Society of Pacing, and Electrophysiology, 1996). Like RMSSD, the HF power mainly depends on parasympathetic activity, being synchronous with respiratory sinus arrhythmia (Task Force of the European Society of Cardiology, and the North American Society of Pacing, and Electrophysiology, 1996). Additionally, the low frequency (LF: 0.04–0.15 Hz) band (which depends on both parasympathetic and sympathetic activity) was analyzed and similarly reported as normalized units (LF_*nu*_) (Task Force of the European Society of Cardiology, and the North American Society of Pacing, and Electrophysiology, 1996). In the non-linear domain, assessment of the short-term (α1) self-similarity coefficient of NN intervals was performed using detrended fluctuation analysis alpha 1 (DFA1) (Peng et al., 1995). The DFA1 indicates changes in the cardiac autonomic modulation, as it increases with a shift in sympathovagal balance toward increased sympathetic drive or lower vagal activity (Tulppo et al., 2001; Penttilä et al., 2003; Castiglioni et al., 2011). It should also be mentioned that DFA1 is not affected by respiratory frequency (Castiglioni et al., 2011) and may thus serve to confirm findings in time- and frequency-domain indices.

#### Performance and Training Experience

The race performance, as well as individual training experience, were assessed through a custom-made questionnaire (see Supplementary Figure S1). Participants were requested to state their total accomplished distance and time, as well as specific reasons for withdrawal before race completion, if applicable (see section “Performance” for details), and their subjectively rated greatest challenge during the *Mammutmarsch*.

Moreover, the questionnaire required participants to provide details about their training experience and fitness level in a standardized form, for example, by reporting the total distance and type of endurance training per week (mentioned here as km/week) in the 6 months prior to the competition. Only kilometers traveled by foot, i.e., walking or running, were considered for the analysis. This data was analyzed to characterize participants’ training status: subjects were divided into two groups based on the median of the weekly training kilometers (i.e., 24.5 km/week) declared for the preceding 6 months. Accordingly, participants were either categorized as highly (HT: ≥24.5 km/week) or poorly trained (PT: <24.5 km/week).

The last section of the questionnaire required participants to subjectively rate their endurance training status at the time of the *Mammutmarsch* on a 10-point Likert scale, with 0 being “extremely poor” and 10 being “excellent.”

### Statistical Analysis

If not otherwise stated, all data are reported as mean ± standard deviation (m ± SD). In total, datasets from 25 competitors were eligible for our statistical analysis. The remaining 31 enrolled athletes were excluded from the analysis due to drop out, insufficient data quality, or loss to follow up (see Figure 1). A sample size of *n* = 25 was deemed appropriate, as research on HRV in ultra-endurance athletes usually accounts for 10–30 subjects (Gratze et al., 2005; Cottin et al., 2007; Foulds et al., 2014; Rundfeldt et al., 2018) and it must be considered that the *Mammutmarsch* as a walking ultramarathon presents a unique study environment.

The entire sample (ALL, *n* = 25) was divided into subgroups. The competitors who reached the 100 km finish line were categorized as “finishers” (FIN), whereas competitors who dropped out before race completion were categorized as “non-finishers” (NON).

Furthermore, standard median split technique was used to assign competitors into dichotomous variables (HT and PT) regarding training status, retrieved from the questionnaires.

The difference of HRV indices due to position change during HR recording (supine vs. standing) was calculated by subtracting per each index the value measured in supine from the value recorded in standing position. The calculated difference was then referred to as “delta” and represented by the correspondent Greek capital letter “Δ” before each HRV index label.

To test for normal distribution, the Shapiro-Wilk-Test was applied, whereas the Equal-Variance-Test served to test for variance. To achieve normal distribution, a log-transformation was administered to RMSSD values (RMSSD_*log*_) (Castiglioni et al., 2011).

Differences between subgroups (e.g., FIN vs. NON) were analyzed using either an unpaired Student’s *t*-test or, when normality check failed, a Mann-Whitney rank sum test.

The relationships between HRV and performance, HRV and training status, as well as between performance and training status were assessed by the analysis of simple linear regression, with the calculation of the Pearson-Product-Moment-Correlation coefficient (*r*) or, if normality was not passed, Spearman Correlation coefficient (*r*_*s*_). Partial correlations controlling for the effect of sex and BMI were computed. All statistical analyses were performed using SigmaPlot 12.3 (Systat Software, San José, CA, United States), with the significance level set at *p* < 0.05.

## Results

### Performance

A total of 25 athletes participating in the 2015 (*n* = 7), 2016 (*n* = 4), and 2018 (*n* = 14) *Mammutmarsch* competitions were included in the statistical analysis. Regarding ALL (*n* = 25), 14 athletes were able to complete the 100 km course (FIN) and 11 competitors withdrew during the race (NON), due to fatigue or musculoskeletal complaints. Table 1 depicts demographic and anthropometric data pre-race. Race performance data is reported in Table 2. In FIN, we observed a slightly reduced velocity compared to NON (*p* = 0.09).

**TABLE 1:** Subject demographics.

	**ALL (*n* = 25; 15 M; 10 W)**	**FIN (*n* = 14; 10 M; 4 W)**	**NON (*n* = 11; 5 M; 6 W)**
**Age, years**			
Men	39 (13)	37 (14)	41 (11)
Women	31 (10)	31 (5)	31 (13)
All	36 (12)	35 (12)	36 (13)
**Weight, kg**			
Men	77 (7)	76 (9)	80 (1)
Women	62(7)*	60 (6)	63 (8)
All	71 (10)	71 (11)	70 (10)
**Height, cm**			
Men	181 (7)	180 (9)	183 (3)
Women	170(6)*	169 (7)	170 (6)
All	177 (9)	177 (10)	176 (8)
**BMI, kg/m^2^**			
Men	23.4 (1.1)	3.2 (1.3)	23.7 (0.5)
Women	21.4(1.2)*	21.1 (0.7)	21.6 (1.4)
All	22.6 (1.5)	22.6 (1.5)	22.6 (1.5)

*S*ubject demographics for all participants (ALL) and subgroups (FIN and NON). No significant differences between FIN and NON groups. *p < 0.05 between women (W) and men (M). [mean (SD)].

**TABLE 2:** Performance data.

	**ALL**	**FIN**	**NON**	***p***
Distance, km	85.4 (17.9)	100.0 (0.0)	66.8 (9.3)	< 0.001
Finish Time, h	18.6 (5.6)	22.7 (2.0)	13.4 (4.1)	< 0.001
Velocity, km/h	4.9 (1.2)	4.4 (0.4)	5.4 (1.7)	0.09

*Performance data [mean (SD)] of all participants (ALL), as well as for subgroups (FIN and NON).*

### Heart Rate Variability

**FIGURE 2:**
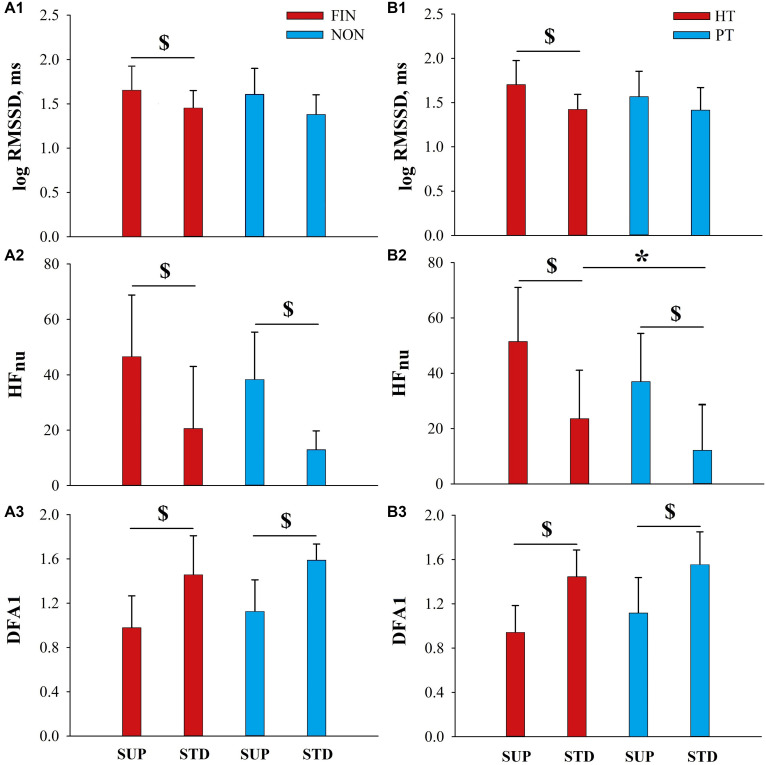
HRV in FIN vs. NON, and HT vs. PT. HRV indices assessed before the race in FIN vs. NON **(A1–A3)**, and HT vs. PT **(B1–B3)**, respectively, in supine (SUP) and standing (STD) position. $ Indicates significant difference between SUP and STD (*p* < 0.05). * Indicates significant difference between HT and PT group for STD-HF_*nu*_ (HT +11.5, *p* = 0.01).

As expected, we found a depressed parasympathetic drive in STD vs. SUP for all participants, irrespective of sex, performance, and training level (Figure 2 and Supplementary Table S1).

No significant differences between men and women were observed in both SUP and STD HRV values, although a tendency to higher vagal modulation was indicated by HF_*nu*_ values in female (SUP: *p* = 0.07; STD: *p* = 0.06 vs. male; Supplementary Table S1). Therefore, data were pooled regardless of sex for further analyses.

Figures 2A1–A3 show HRV indices in FIN and NON groups. No significant differences in FIN vs. NON for HRV parameters could be detected.

**FIGURE 3:**
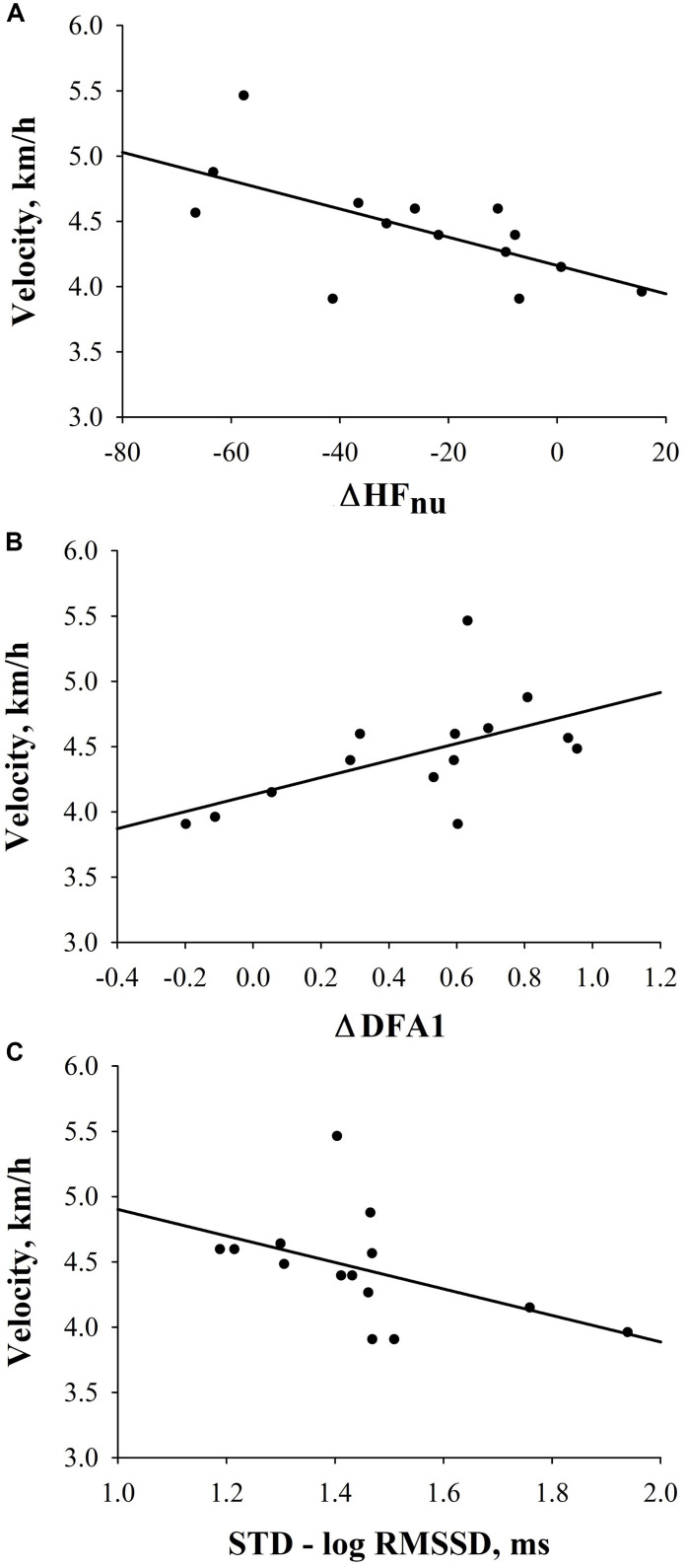
Correlations between HRV indices and velocity in FIN. **(A)** Correlation between ΔHF_*nu*_ and velocity (*r* = −0.7; *p* = 0.01). **(B)** Correlation between ΔDFA1 and velocity (*r* = 0.6; *p* = 0.04). **(C)** Correlation between STD-RMSSD_*log*_ and velocity (*r* = −0.5; *p* = 0.08).

Interestingly, in FIN, a significant negative correlation between ΔHF_*nu*_ and velocity [velocity = 4.162 - (0.0108 × ΔHF_*nu*_); *r* = −0.65, *p* = 0.01)] (i.e., the higher the decrease from SUP- to STD-HF_*nu*_, the higher the velocity), as well as a significant positive correlation between ΔDFA1 [velocity = 4.132 + (0.652 × ΔDFA1); *r* = 0.57, *p* = 0.04)] and velocity (i.e., the higher the increase to STD-DFA1, the higher the velocity), were retrieved (Figures 3A,B). Conversely, none of the investigated HRV indices were significantly correlated with performance in NON. Furthermore, in FIN, there was a trend to a negative correlation between STD-RMSSD_*log*_ and velocity [velocity = 5.919 - (1.017 × STD-RMSSD); *r* = −0.48, *p* = 0.08); Figure 3C]. Supplementary Table S2 further depicts correlations between pre-race HRV and performance in FIN and NON groups. Similar results were obtained when computing partial correlations controlling for the effect of sex and BMI.

### Training Experience

Training experience data was retrieved for 24 competitors, as one athlete was not available for post-race follow-up. Assessment of subjective records served to characterize the training volume (mean km/week during the past 6 months prior to competition), as well as self-rated endurance capacity, as “training status.” In ALL, the mean distance walked or run per week amounted to 30 ± 23 km (ranging from 5 to 112.5 km/week). The subjectively perceived training status among competitors was rated at 7.5 ± 1.4 out of 10 (Supplementary Table S3).

**FIGURE 4:**
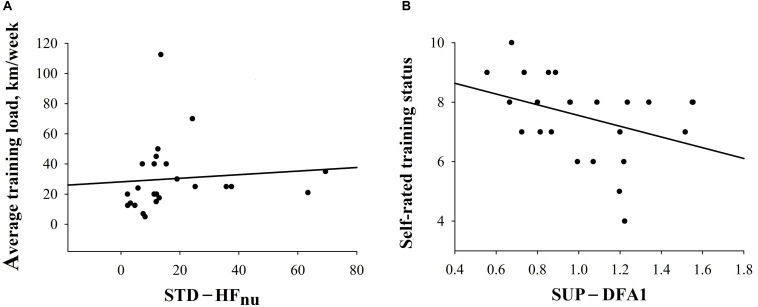
Correlations between HRV indices and training experience in ALL. **(A)** Correlation between STD-HF_*nu*_ and km/week (*r* = 0.5; *p* = 0.01). **(B)** Correlation between SUP-DFA1 and self-reported endurance training status (*r* = −0.4; *p* = 0.07).

Additionally, the relationship between training experience and pre-race HRV, as well as performance, was investigated in the entire sample. In ALL, a higher STD-HF_*nu*_ would significantly relate to greater km/week [STD-HF_*nu*_ = 15.750 + (0.0692 × km/week); *r*_*s*_ = 0.50, *p* = 0.01)] and a lower SUP-DFA1 was associated with higher self-rated endurance status (SUP-DFA1 = 1.616 - (0.0783 × training status); *r*_*s*_ = −0.38, *p* = 0.07)], although this did not attain significance (Figure 4 and Supplementary Table S4). There were no significant correlations between training experience (both km/week and self-reported training status) and performance (velocity, total distance covered) in ALL.

As mentioned, split median analysis of reported training volume (km/week) served to assign competitors to highly (HT, *n* = 12) and poorly trained (PT, *n* = 12) subgroups. HT presented a significantly higher training volume (+29 km/week, *p* < 0.001). However, subjectively rated endurance capacity did not differ between groups (Supplementary Table S3 for subgroup details).

Pre-race HRV values in HT and PT groups are depicted in Figures 2B1–B3. Greater parasympathetic drive in HT compared to PT was indicated by significantly higher STD-HF_*nu*_ (+12, *p* = 0.01), whereas STD-LF_*nu*_ was lower in HT vs. PT (-12, *p* = 0.01), in line with above-mentioned findings in ALL.

**FIGURE 5:**
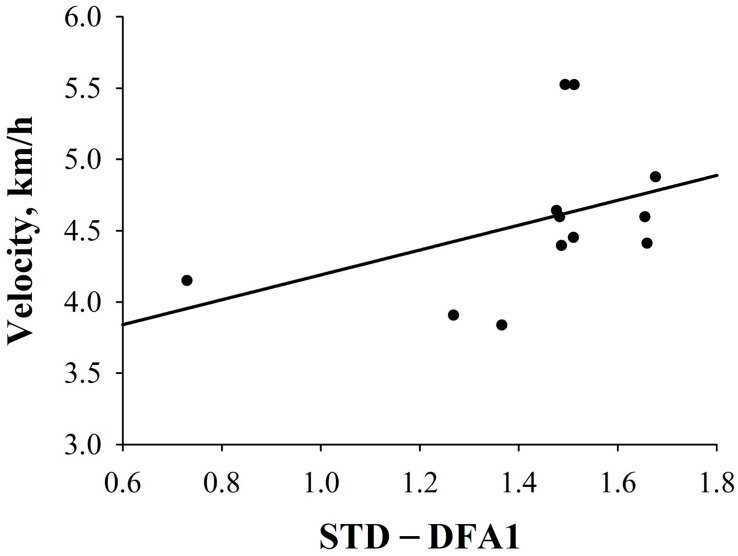
Correlations between HRV indices and performance in HT. Correlation between STD-DFA1 and velocity (km/h) (*r* = 0.6; *p* < 0.05).

Furthermore, in HT, a higher DFA1 value in STD predicted a significantly greater velocity [velocity = 3.316 + (0.873 × STD-DFA1); *r*_*s*_ = 0.57, *p* = 0.04; Figure 5]. In PT, no significant correlations between HRV indices and performance could be observed. Supplementary Table S5 depicts results of correlation analysis between pre-race HRV and performance in HT and PT groups.

## Discussion

This study was conducted to evaluate the relationship between short-term baseline HRV indices, and both performance and training experience, in a group of amateur athletes participating in a 100 km walking ultramarathon. Our findings emphasize the value of pre-race HRV assessment for performance prediction in ultra-endurance competitions. Moreover, regarding the use of HRV as a tool to monitor and predict performance, our results support the additional value of including an orthostatic challenge (supine-standing), compared to HRV assessment exclusively in supine position. We could not observe any sex-related differences in supine HRV. Current research in standard populations demonstrated women to account for greater parasympathetic drive compared to men (Koenig and Thayer, 2016). However, HRV may also depend on body composition (Esco et al., 2011; Koenig et al., 2014), physical activity (Rennie et al., 2003), and, in females, on the phase of the menstrual cycle (Schäfer et al., 2015), whose analysis is beyond the purpose of this study. Unfortunately, the proportion of female ultramarathoners is still smaller compared to male athletes, and a great number of studies investigated HRV parameters in male ultra-endurance athletes only, or accounted for very few female subjects (Gratze et al., 2005; Cottin et al., 2007; Ramos-Campo et al., 2019). Further studies with larger female samples are therefore warranted.

The hypothesis that successful competitors (FIN) would be characterized by higher parasympathetic activity in pre-race measurements compared to non-finishing athletes (NON) was not supported by the results. This can be due to several aspects, such as, for example, specific race characteristics. The *Mammutmarsch* must be performed by *walking* only and *running* is not allowed. This creates a unique study environment and, to our knowledge, only one other study investigated cardiac autonomic modulation in a 24-h walking competition so far (Cottin et al., 2007). Also, in a walking amateur ultramarathon, extensive endurance training and preparation may not be required in order to compete. This is in contrast with other highly demanding ultramarathons, such as the Yukon Arctic Ultra, which is performed on a 690 km course in a subarctic climate (Rundfeldt et al., 2018).

Regarding performance, FIN accounted for a slower (by 1 km/h) velocity compared to NON. Even if this did not attain significance (*p* = 0.09), it may provide a valid argument to speculate about the characteristics of successful pacing, especially in this setting of a *walking ultramarathon*. Indeed, in ultramarathon, pacing may be essential (Knechtle et al., 2019). Improved performance has been associated with a reduction in initial speed (after reaching peak velocity, i.e., positive pacing) and lower variability in velocity (Bernard et al., 2009; Matta et al., 2019). Bossi et al. observed the fastest and most successful 24-h ultramarathon competitors to account for lower starting velocities, compared to less successful athletes (Bossi et al., 2017), this underlines our finding of lower overall velocity in FIN. Nevertheless, specific characteristics of pacing in a walking ultramarathon remain beyond the scope of this work and further studies are warranted.

To date, fewer investigations on the relationship between HRV and its change during orthostatic challenge, and ultra-endurance competition performance have been conducted (Bernardi et al., 1997; Gratze et al., 2005; Vescovi, 2019). Previous investigations reported the value and reliability of HRV assessment during standing in athletes (Boullosa et al., 2014). An orthostatic provocation maneuver may reflect autonomic dynamic adaptation, with a shift in cardiac autonomic modulation toward increased sympathetic and decreased parasympathetic drive (Hynynen et al., 2011; Schäfer et al., 2015), as shown, respectively, by higher DFA1 (Tulppo et al., 2001) and decreased HF power (Gonçalves et al., 2015). Indeed, HRV may be described as “a psychophysiological measure of autonomic flexibility” (Williams et al., 2019), indirectly reflecting the ability to respond to various internal and external demands (An et al., 2020). Conversely, a reduced autonomic responsiveness to stressors, such as an excitatory stimulus during orthostatic challenge, has been proposed as the most common feature of pathophysiological conditions (Grant et al., 2012). Gratze et al. observed that, after ultra-endurance competition, the adaptability to orthostatism is dramatically reduced, as many athletes experience orthostatic intolerance (Gratze et al., 2005). Therefore, the ability of the autonomic and cardiovascular system to react to a marked and persistent reduction of blood pressure (BP), which occurred immediately after ultra-endurance performance (Gratze et al., 2005), is vital to limit the cardiac effort necessary to counteract the BP fall. Implementing an orthostatic challenge (during which BP is abruptly reduced) (Ueno and Moritani, 2003) may thus provide information on the autonomic ability to react to (and counteract) BP falls immediately after endurance exercise, which is currently considered one of the main reasons of cardiovascular fatigue in endurance athletes (Gratze et al., 2005). This may be mitigated by optimal training, as a more pronounced decrease in parasympathetic drive (and thus possible “preservation” of orthostatic tolerance) after standing up has been observed in women with higher training volumes compared with those with lower training volumes (Gilder and Ramsbottom, 2008). There is further evidence showing that the changes in cardiac autonomic modulation during orthostatic stress may better reflect the relationship between HRV (in terms of parasympathetic responsiveness) and physical capacity and activity (Ueno and Moritani, 2003; Grant et al., 2009; Gonçalves et al., 2015), as well as exercise volume (Gilder and Ramsbottom, 2008) and training load (or overtraining) (Hynynen et al., 2008), as orthostasis may sensitively depict exercise-induced changes in cardiac autonomic modulation in response to a stimulus (Grant et al., 2012; Schneider et al., 2019).

Only in FIN group, significant correlations between HRV and race performance were observed: a greater decrease in vagal drive upon position change would predict a greater velocity. Firstly, only the transition between supine and standing postures (i.e., Δvalues) could reflect the association between HRV and performance. Secondly, we obtained two similar significant regressions by examining two HRV indices from two different analysis domains: HF_*nu*_ is a spectral index (Task Force of the European Society of Cardiology, and the North American Society of Pacing, and Electrophysiology, 1996) and the DFA1 is a recursive index of signal complexity (Castiglioni et al., 2011). Therefore, these findings are unlikely to derive from a simply casual observation. Generally, aerobic training chronically increases vagal drive (Iellamo et al., 2004; Buchheit, 2014). However, in training periodization, parasympathetic drive may decrease pre-competition, while sympathovagal drive increases and predominates (Iellamo et al., 2002; Manzi et al., 2009; Hug et al., 2014). According to previous research, autonomic responsiveness (i.e., parasympathetic withdrawal reactive to orthostatic challenge, representing an essential marker of healthy autonomic reactivity) (Grant et al., 2012) may relate to both physical activity and training load (Grant et al., 2012) as well as physical capacity (e.g., VO_2max_) (Gilder and Ramsbottom, 2008). In return, training volume (Knechtle et al., 2010) and physical capacity (Millet et al., 2011) have both been associated with greater endurance performance. It is likely that the capacity to override vagal predominance chronically acquired by long-term aerobic training may then facilitate race performance (Iellamo et al., 2002). This underlines our suggestion that FIN may account for a better dynamic ability (i.e., parasympathetic responsiveness) (Grant et al., 2012) to adapt cardiac autonomic modulation to a physiological challenge and therefore be more successful. Indeed, in NON, no correlations between HRV and performance could be observed. Although NON did account for similar resting HRV like FIN, they may not have been able to significantly overdrive parasympathetic predominance during orthostatic challenge, in order to allow successful prolonged cardiac performance. Still, the description of the physiological mechanisms underlying the observed phenomenon is beyond the scope of this paper and deserve further investigations.

Furthermore, the HT group showed greater vagal drive than competitors with lower training volume, the PT group (Figure 2B3 and “Training Experience”), as confirmed by correlation analysis between HRV and training experience in the entire sample of athletes (ALL) (Figure 4 and Supplementary Table S4). Specifically, we observed greater training volume and higher subjectively rated endurance capacity to be associated with higher indices of vagal modulation. As for subjective training status, we observed higher endurance capacity ratings in participants with lower DFA1 (i.e., greater vagal modulation) during supine rest, in line with previous findings (Ravé et al., 2020). Also, the observed positive correlations between greater vagal drive and higher training volume are in line with current findings of higher parasympathetic predominance and greater total HRV at rest in response to prolonged aerobic exercise (Hautala et al., 2001; Bellenger et al., 2016). Therefore, the subjective training experience assessment in amateur athletes through post-race questionnaires seems to be a valuable and feasible tool, as it may be difficult to monitor training over an extended time period in leisure-time ultramarathon competitors.

### Limitations

Although we deemed the total sample size of *n* = 25 to be appropriate (see section “Statistical Analysis”), higher sample numbers are warranted. In addition, future studies should implement larger female sample proportions, as women are generally underrepresented in ultramarathon research (Gratze et al., 2005; Cottin et al., 2007; Ramos-Campo et al., 2019). As for HRV assessment methods, we did not record respiration rate or blood pressure. Both may influence HRV during supine and standing (Brown et al., 1993), while blood pressure may especially mediate HRV parameter changes in response to orthostatic challenge (Task Force of the European Society of Cardiology, and the North American Society of Pacing, and Electrophysiology, 1996; Ueno and Moritani, 2003). Finally, we could not assess a possible influence of body composition on cardiac autonomic modulation (Esco et al., 2011; Koenig et al., 2014), as we did not measure body fat. Regarding training experience assessment, although post-race self-reported measures resulted as a valuable and feasible tool, controlled assessment of training experience may have generated more accurate data.

## Conclusion

In trained and successful amateur ultramarathoners, higher pre-race vagal drive may be associated with better training experience and predict competition success. However, in poorly trained and unsuccessful competitors, supine HRV assessment may not serve for performance prediction. Additionally, HRV assessment throughout an orthostatic challenge may provide additional information on dynamic adaptability of the ANS to physiologic challenges and therefore be valuable for performance prediction. Self-reported training experience may be reliably associated with parasympathetic drive, therefore indirectly predicting performance.

## Data Availability Statement

The datasets generated for this study are available on request to the corresponding author.

## Ethics Statement

The studies involving human participants were reviewed and approved by the Ethics Committee of the Charité – Universitätsmedizin Berlin, Germany (document number EA1/163/14). The patients/participants provided their written informed consent to participate in this study.

## Author Contributions

MM and LR contributed equally to the study by writing the manuscript and analyzing the data. GM contributed equally with MS by designing the study, structuring data results, and drafting the manuscript. MS planned and implemented the study with the help of MJ, LR, and MM. H-CG and GM provided critical expertise, feedback, and revised with all the co-authors the final manuscript. All authors contributed to the article and approved the submitted version.

## Conflict of Interest

The authors declare that the research was conducted in the absence of any commercial or financial relationships that could be construed as a potential conflict of interest.
