# Supplementary material for: The Advantage of Supine and Standing Heart Rate Variability Analysis to Assess Training Status and Performance in a Walking Ultramarathon
Source: Front Physiol. 2020 Jul 24;11:731. doi: 10.3389/fphys.2020.00731 (PMC7394006; doi:10.3389/fphys.2020.00731)
Supplement: Supplementary file 1 [file Table_1.DOCX]

**Supplementary Table S1 |** HRV in male vs. female.

| **Parameter** | **Recording  position** | male (n = 15)  mean (S.D.) | female (n = 10)  mean (S.D.) | *p* |
| --- | --- | --- | --- | --- |
| **RMSSD_log_** | SUP | 1.6 (0.3) | 1.7 (0.3) | *0.25* |
|  | STD | 1.4 (0.2) | 1.4 (0.2) $ | *0.64* |
| **HF_nu_** | SUP | 36.9 (19.8) | 51.9 (18.3) | *0.07 §* |
|  | STD | 12.6 (15.0) $ | 24.2 (19.5) $ | *0.06 §* |
| **DFA1** | SUP | 1.1 (0.3) | 1.0 (0.2) | *0.39* |
|  | STD | 1.6 (0.3) $ | 1.5 (0.3) $ | *0.33* |

*HRV indices assessed before the race in male vs. female, respectively in supine (SUP) and standing (STD) position. $ Indicates significant difference between SUP and STD. No significant differences between male and female groups. § Indicates trend to significant difference between male and female groups (p < 0.1 > 0.05).*
