# Supplementary material for: The Advantage of Supine and Standing Heart Rate Variability Analysis to Assess Training Status and Performance in a Walking Ultramarathon
Source: Front Physiol. 2020 Jul 24;11:731. doi: 10.3389/fphys.2020.00731 (PMC7394006; doi:10.3389/fphys.2020.00731)
Supplement: Supplementary file 2 [file Table_2.DOCX]

**Supplementary Table S2 |** Correlations between HRV and performance in FIN and NON.

| **FIN** | **Recording  position** | mean velocity  (km/h) | **NON** | **Recording  position** | mean velocity  (km/h) |
| --- | --- | --- | --- | --- | --- |
| **RMSSD_log_** | SUP | r -0.01 | **RMSSD_log_** | SUP | r -0.02 |
|  | STD | r -0.48 § |  | STD | r 0.46 |
|  | Δ | r -0.40 |  | Δ | r 0.35 |
| **HF_nu_** | SUP | r 0.23 | **HF_nu_** | SUP | r -0.25 |
|  | STD | r -0.30 |  | STD | r -0.43 |
|  | Δ | r -0.65 * |  | Δ | r 0.32 |
| **DFA1** | SUP | r -0.19 | **DFA1** | SUP | r 0.27 |
|  | STD | r 0.40 |  | STD | r 0.15 |
|  | Δ | r 0.57 * |  | Δ | r -0.35 |

*Correlations between HRV indices assessed before the race, respectively, for supine (SUP) and standing (STD) position, as well as delta value, and velocity (km / h), for FIN and NON groups. * Indicates significant correlation (p < 0.05). § Indicates trend to significant correlation
(p < 0.1 > 0.05).*
