# Supplementary material for: The Advantage of Supine and Standing Heart Rate Variability Analysis to Assess Training Status and Performance in a Walking Ultramarathon
Source: Front Physiol. 2020 Jul 24;11:731. doi: 10.3389/fphys.2020.00731 (PMC7394006; doi:10.3389/fphys.2020.00731)
Supplement: Supplementary file 3 [file Table_3.DOCX]

**Supplementary Table S3 |** Training Experience.

| Parameter | Gender | **ALL**  (n = 25;  m = 15; f = 10) | **HT**  (n = 12;  m = 10; f = 4) | **PT**  (n = 12;  m = 5; f = 6) | *p  (HT vs. PT)* |
| --- | --- | --- | --- | --- | --- |
| km/week  mean (S.D.) | **Men** | 30 (27) | 59 (36) | 18 (4) |  |
|  | **Women** | 31 (19) | 38 (15) | 6 (1) |  |
|  | **All** | 30 (23) | 45 (25) | 16s (6) | *< 0.001* |
| self-rated training status  mean (S.D.) | **Men** | 7.7 (0.8) | 8.0 (0.8) | 7.6 (0.8) |  |
|  | **Women** | 7.2 (1.9) | 7.4 (2) | 6.5 (2.1) |  |
|  | **All** | 7.5 (1.4) | 7.6 (1.7) | 7.4 (1.1) | *0.78* |

*Total distance of endurance training per week (km/week) and self-rated endurance training status in the entire sample (ALL), as well as highly (HT) and poorly trained (PT) groups.
* Indicates significant difference between HT (All) and PT (All) groups (p < 0.05).*
