# Supplementary material for: The Advantage of Supine and Standing Heart Rate Variability Analysis to Assess Training Status and Performance in a Walking Ultramarathon
Source: Front Physiol. 2020 Jul 24;11:731. doi: 10.3389/fphys.2020.00731 (PMC7394006; doi:10.3389/fphys.2020.00731)
Supplement: Supplementary file 4 [file Table_4.DOCX]

**Supplementary Table S4 |** Correlations between HRV and training
experience in ALL.

| **ALL** | **Recording  position** | km/week | self-rated  training status |
| --- | --- | --- | --- |
| **RMSSD_log_** | SUP | r 0.21 | r 0.09 |
|  | STD | r 0.06 | r -0.21 |
|  | Δ | r -0.12 | r -0.31 |
| **HF_nu_** | SUP | r 0.19 | r 0.27 |
|  | STD | r 0.50 * | r 0.11 |
|  | Δ | r < 0.001 | r -0.06 |
| **DFA1** | SUP | r -0.18 | r -0.38 § |
|  | STD | r -0.25 | r -0.20 |
|  | Δ | r -0.02 | r 0.16 |

*Correlations between HRV indices assessed before the race, respectively, for supine (SUP) and standing (STD) position, as well as Δ-value, and total distance of endurance training per week (km/week), as well as self-rated endurance training status, in the entire sample (ALL).
* Indicates significant correlation (p < 0.05). § Indicates trend to significant correlation
(p < 0.1 > 0.05).*
