# Supplementary material for: The Advantage of Supine and Standing Heart Rate Variability Analysis to Assess Training Status and Performance in a Walking Ultramarathon
Source: Front Physiol. 2020 Jul 24;11:731. doi: 10.3389/fphys.2020.00731 (PMC7394006; doi:10.3389/fphys.2020.00731)
Supplement: Supplementary file 5 [file Table_5.DOCX]

**Supplementary Table S5 |** Correlations between HRV and performance in HT and PT.

| **HT** | **Recording  position** | mean velocity (km/h) | **PT** | **Recording  position** | mean velocity  (km/h) |
| --- | --- | --- | --- | --- | --- |
| **RMSSD_log_** | SUP | r -0.28 | **RMSSD_log_** | SUP | r 0.05 |
|  | STD | r -0.04 |  | STD | r -0.06 |
|  | Δ | r 0.38 |  | Δ | r -0.10 |
| **HF_nu_** | SUP | r -0.23 | **HF_nu_** | SUP | r 0.19 |
|  | STD | r -0.42 |  | STD | r -0.42 |
|  | Δ | r -0.11 |  | Δ | r -0.42 |
| **DFA1** | SUP | r 0.38 | **DFA1** | SUP | r -0.02 |
|  | STD | r 0.57 * |  | STD | r 0.15 |
|  | Δ | r 0.06 |  | Δ | r 0.29 |

*Correlations between HRV indices assessed before the race, respectively, for supine (SUP) and standing (STD) position, as well as Δ-value, and velocity (km / h), for HT and PT group. * Indicates significant correlation (p < 0.05).*
