# Supplementary figures and images for: The Advantage of Supine and Standing Heart Rate Variability Analysis to Assess Training Status and Performance in a Walking Ultramarathon
Source: Front Physiol. 2020 Jul 24;11:731. doi: 10.3389/fphys.2020.00731 (PMC7394006; doi:10.3389/fphys.2020.00731)

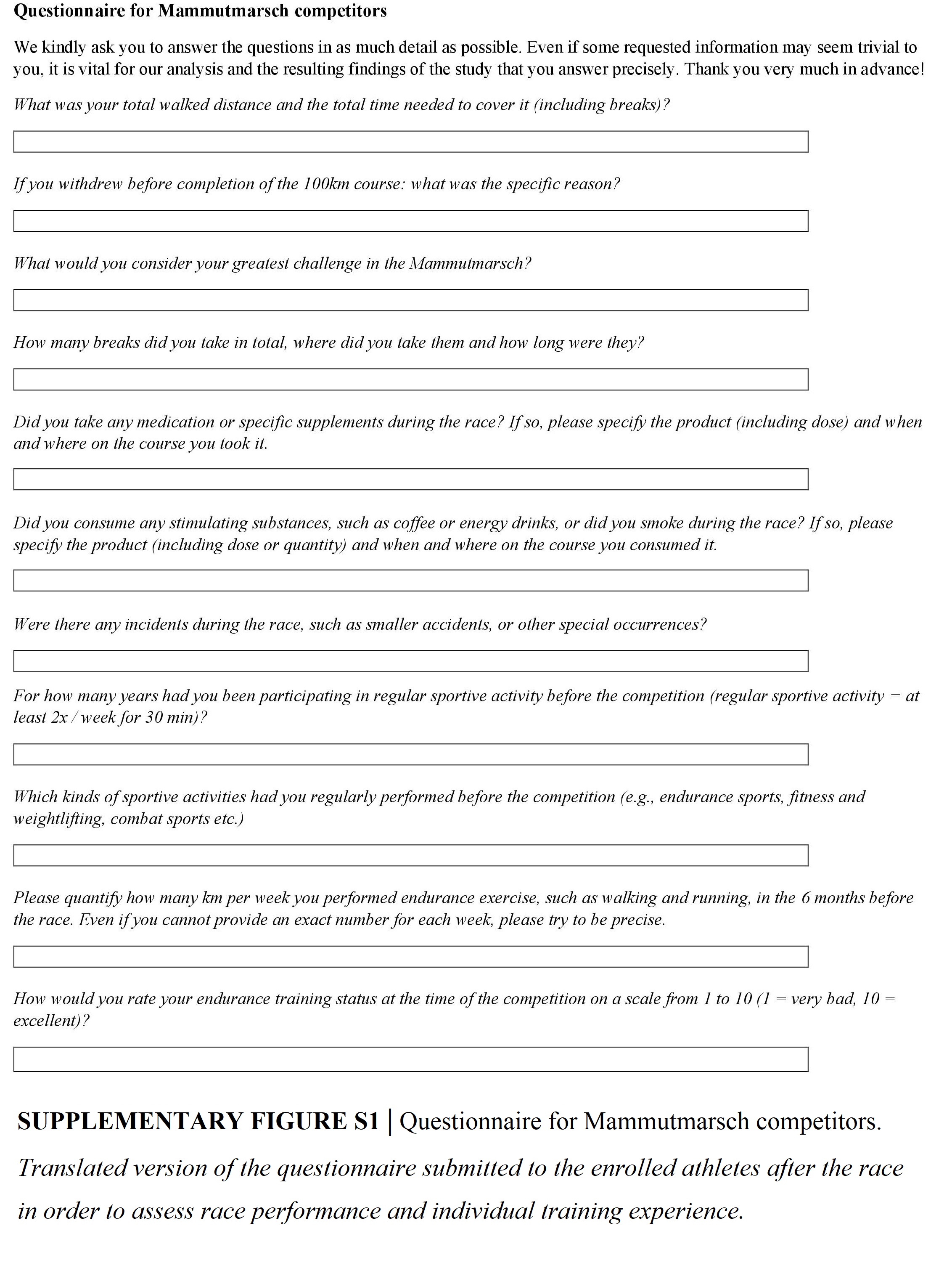

Supplement: Supplementary file 6 [file Image_1.JPEG]
